# Cross-Species Insights into *In Vitro* Maturation Defects of the Oocyte and Identification of Crucial Regulators for Sheep Oocyte Maturation

**DOI:** 10.3390/antiox14121499

**Published:** 2025-12-13

**Authors:** Jian Cui, Xiurong Zhao, Jia Hao, Xingyuan Liu, Wenjing Wang, Lixia He, Yubing Wang, Jinfu Rong, Chunjuan Qiu, Dayong Chen, Lei Cheng, Jianhui Tian, Jiaxin Zhang, Guangyin Xi

**Affiliations:** 1 Key Laboratory of Animal Genetics, Breeding and Reproduction of the Ministry of Agriculture and Rural Affairs, Frontiers Science Center for Molecular Design Breeding (MOE), College of Animal Science and Technology, China Agricultural University, Beijing 100193, China; b20223040316@cau.edu.cn (J.C.); bs20213040375@cau.edu.cn (X.Z.); hhj7653@cau.edu.cn (J.H.); s20233040734@cau.edu.cn (X.L.); wangwenjing@sdau.edu.cn (W.W.); b20243040418@cau.edu.cn (Y.W.); tianjh@cau.edu.cn (J.T.); 2 Inner Mongolia Key Laboratory of Sheep & Goat Genetics Breeding and Reproduction, College of Animal Science, Inner Mongolia Agricultural University, Hohhot 010018, China; hlx99886@emails.imau.edu.cn (L.H.); 970688907@emails.imau.edu.cn (J.R.); 3 Inner Mongolia Sino Sheep Technology Co., Ltd., Ulanqab 011800, China; 18247412186@163.com (C.Q.); chendayong81@126.com (D.C.); cl344713520@163.com (L.C.)

**Keywords:** oocyte, *in vitro* maturation, transcriptomics, endoplasmic reticulum stress, EFNA1, NRXN1

## Abstract

The poor quality of oocytes matured *in vitro* seriously hinders the application in mammalian assisted reproductive technology (ART). Exploring the regulators and mechanisms influencing oocyte maturation is critical to improve the developmental competence of *in vitro* matured oocytes and the efficiency of ART. Here, through comparative cross-species transcriptomic analyses, we reveal that impaired autocrine/paracrine signaling and disruption of ubiquitin-dependent protein catabolic process, which are often accompanied by severe endoplasmic reticulum stress, represent common potential defects during *in vitro* oocyte maturation. Moreover, we identified two previously unrecognized key factors missing in the current IVM system by ligand screening. We further determined that EFNA1 and NRXN1 alleviated the excessive accumulation of protein aggregates and endoplasmic reticulum stress by enhancing the oocyte antioxidant defense and maintaining lipid homeostasis, thereby improving the oocyte developmental potential. Our findings identified critical extrinsic regulators of oocyte developmental competence and provided a practical strategy to improve IVM efficiency in ART.

## 1. Introduction

Oocyte *in vitro* maturation (IVM) serves as a critical component of assisted reproductive technologies (ART) in both humans and farm animals, as it generates oocytes capable of fertilization *in vitro* and yields transferable embryos [1,2,3]. IVM is important both for human IVF in cases where ovarian stimulation is contraindicated and for animal IVF to accelerate *in vitro* embryo production [4,5,6]. However, oocytes matured *in vitro* show inferior outcomes in maturation quality, subsequent embryo developmental potential, implantation competence, and pregnancy success compared to those matured *in vivo* [7,8,9,10,11]. Current IVM systems inadequately mimic the *in vivo* microenvironment, leading to elevated reactive oxygen species (ROS) from mitochondrial dysfunction and abnormal lipid accumulation in oocytes [12,13]. This oxidative and lipotoxic stress triggers endoplasmic reticulum (ER) stress in multiple species (including humans, mice, and cattle), ultimately impairing developmental potential as a direct result of non-physiological maturation conditions [14,15,16,17]. Consequently, optimizing the microenvironment of oocyte maturation *in vitro* is imperative to enhance the quality of oocyte maturation and developmental potential.

*In vivo*, oocyte growth and maturation are inextricably coupled to the nurturing microenvironment of the follicle, with indispensable support provided by the somatic cell compartment, primarily cumulus cells [18]. Such exquisitely regulated ligand–receptor dynamics, mediated through both autocrine and paracrine signaling between oocytes and cumulus cells, are fundamentally prerequisite for establishing comprehensive oocyte developmental competence. For example, oocyte-secreted factors, BMP15 and GDF9, play pivotal roles in enhancing oocyte developmental competence and promoting cumulus cell function [19,20]. EGF-like peptides, including epiregulin and amphiregulin, are secreted in parallel by cumulus cells and function as pivotal regulators of both oocyte maturation and subsequent embryo quality [21,22]. Thus, a comprehensive elucidation of the ligand–receptor networks that mediate bidirectional communication between the oocyte and its surrounding cumulus cells within the follicular microenvironment is essential for optimizing *in vitro* oocyte maturation systems. To date, a repertoire of well-characterized ligands secreted by oocytes and cumulus cells has been incorporated into human, ovine, bovine, and porcine IVM medium to improve oocyte quality [20,23,24,25]. Nevertheless, robust and scalable platforms for systematically screening yet undiscovered ligands with oocyte-quality-promoting potential remain conspicuously absent in both human and large domestic species.

Recent advances in single-cell RNA sequencing (scRNA-seq) technology and the expansion of protein–protein interaction repositories have made it routine to interrogate intercellular signaling directly from single-cell gene expression profiles [26]. Notably, ligand–receptor pair analysis has emerged as a particularly powerful framework for reconstructing cell–cell communication networks [27]. This paradigm is operationalized through dedicated computational pipelines, including CellPhoneDB v2.0, CellTalkDB, and CellCall, which systematically quantify putative signaling events by integrating ligand and cognate receptor expression with structural interaction data [28,29,30,31]. Leveraging CellPhoneDB v2.0 in conjunction with high-resolution scRNA-seq therefore affords a robust, unbiased strategy for identifying physiologically relevant ligands that govern oocyte maturation in humans and large domestic species.

Capitalizing on this technological synergy, we employed sheep as a translational model to dissect the molecular underpinnings of the developmental competence gap between *in vivo* and *in vitro* matured oocytes, aiming to inform the rational refinement of IVM protocols. Through comparative cross-species transcriptomic analysis of *in vivo* and *in vitro* matured oocytes, we identified that the abnormality of ubiquitin-dependent protein catabolic process and autocrine/paracrine signaling in oocyte might be the important causes for the reduction in quality of matured oocytes *in vitro*. By applying CellPhoneDB v2.0 to transcriptomes of paired cumulus cells and oocytes, we delineated the complete ligand–receptor interactome active during both maturation contexts. This analysis revealed a discrete subset of ligands whose signaling intensities were markedly enhanced under *in vivo* conditions. Within this signature, we newly implicate EFNA1 and NRXN1 as regulators of oocyte maturation, expanding the catalog of factors capable of steering developmental competence. Our integrative approach thus provides a scalable platform for discovering previously unrecognized oocyte maturation regulators and offers immediate translational avenues for clinical and agricultural enhancement of oocyte quality.

## 2. Materials and Methods

### 2.1. Oocyte In Vitro Maturation, Fertilization, and Embryo In Vitro Culture

Sheep ovaries collected from a local abattoir were transported to the laboratory within 3–4 h at 30–35 °C in 0.9% NaCl solution. Upon arrival, ovaries were washed three times in 37 °C saline solution supplemented with 100 IU/mL penicillin and 100 IU/mL streptomycin, cumulus-oocyte complexes (COCs) were aspirated from 3 to 6 mm follicles using a 20-gauge needle. Only oocytes with homogeneous cytoplasm and compact cumulus cell layers were selected for IVM. Approximately 30 COCs were cultured per well in 100 μL maturation medium for 24 h at 38.5 °C under 5% CO_2_ in humidified air. Following *in vitro* maturation (IVM), cumulus cells were removed by vortexing for 30 s in 0.1% (*w*/*v*) hyaluronidase solution. Mature oocytes were then transferred to 100 μL droplets of fertilization medium (synthetic oviductal fluid [SOF] supplemented with 10% estrous sheep serum) and co-incubated with frozen-thawed sperm (1 × 10^6^/mL) for 19–21 h. Presumptive zygotes were washed and cultured in SOF supplemented with 3 mg/mL bovine serum albumin (BSA) under mineral oil at 38.5 °C in a humidified atmosphere of 5% CO_2_, 5% O_2_, and 90% N_2_ until day 7.

### 2.2. Collection of Oocytes, Cumulus Cells, and Mural Granulosa Ccells Under Different Maturation Conditions

A total of 45 healthy adult Mongolian ewes (2–5 years) were maintained on a diet of high-quality hay supplemented with concentrate (0.5 kg/head/day) provided twice daily with ad libitum water. Estrus synchronization was achieved using intravaginal CIDR^®^ devices (Pharmacia & Upjohn, Hartwell, Australia) with two equal FSH doses (Sansheng, Ningbo, China) administered on days 10 and 11 [32].

All GV-stage oocytes used in this study (for both subsequent *in vivo* and *in vitro* maturation) were collected from live ewes via Laparoscopic Ovum Pick-Up (LOPU). For *in vivo* matured oocytes, CIDRs were withdrawn on day 12, after which the ewes received an LHRH-A3 injection (Sansheng, Ningbo, China) on day 13, and oocytes were collected by oviduct flushing 26 h later using DPBS containing 1 mg/mL BSA and 0.7 mg/mL heparin. For IVM, GV oocytes were collected by LOPU after CIDRs withdrawal and then cultured *in vitro* for 24 h to produce mature (MII) oocytes surrounded by their accompanying cumulus cells. Follicles meeting size criteria were selected, and follicular contents including oocytes and surrounding somatic cells were aspirated via LOPU. COCs were mechanically dissociated using 29G needles (Shuguang, Henan, China) to separate denuded oocytes from their cumulus cell investments while preventing cross-contamination. For MGCs isolation, large sheet-like granulosa cell aggregates were selectively collected for sequencing. During the LOPU procedure, follicular fluid was aspirated from each donor ewe. From this aspirate, three distinct cell populations—oocytes, cumulus cells (CCs), and mural granulosa cells (MGCs)—were isolated and processed independently as separate sequencing samples.

All procedures and protocols involving sheep were approved by the Animal Ethics Committee of China Agricultural University (AW12805202-1-01) on 21 August 2025.

### 2.3. Assessment of the Nuclear Maturation and Blastocyst Cell Number

After IVM, COCs were treated with 0.1% hyaluronidase and vortexed for 30 s. Oocytes were then fixed in 4% paraformaldehyde (PFA) for 15 min at room temperature, stained with Hoechst 33342 (C1026, Beyotime, Jiangsu, China) for 10 min, and nuclear maturation status was evaluated by a fluorescence microscope. Day 7 blastocysts were fixed in 4% PFA and stained with Hoechst 33342 to determine the total cell number by using Image J software (ver. 1.54i).

### 2.4. Measurement of Intracellular ROS and GSH Levels

ROS and GSH levels were evaluated using the ROS assay kit (S0033S, Beyotime, Jiangsu, China) and CMF2HC (HY-D1571, MedChemexpress, Princeton, NJ, USA). After IVM, COCs were treated with 0.1% hyaluronidase and vortexed for 30 s. Oocytes from each experimental group were incubated in darkness for 30 min at 38.5 °C under 5% CO_2_ in IVM medium containing 10 μM 2′,7′-dichlorodihydrofluorescein diacetate (H2DCFDA) for ROS detection and 10 μM CellTracker™ Blue for GSH measurement. Subsequently, the oocytes were washed three times in PBS-PVA. Fluorescence signals were captured using fluorescence microscopy (Nikon, Tokyo, Japan). Analysis was performed by Image J software.

### 2.5. Measurement of Lipid Droplet and Fatty Acid

Lipid droplet and fatty acid analysis were performed as described in the manufacturer’s instructions. After IVM, COCs were treated with 0.1% hyaluronidase and vortexed for 30 s, fixed in 4% PFA for 30 min. The fixed oocytes were washed three times in PBS-PVA and incubated with 10 μg/mL Nile Red (C2051S, Beyotime, Jiangsu, China) and 10 μg/mL BODIPY 500/510 C1, C12 (C2055, Beyotime, Jiangsu, China) for 1 h at room temperature. Subsequently, the oocytes were washed three times in PBS-PVA. Fluorescence signals were captured using fluorescence microscopy (Nikon, Tokyo, Japan). Analysis was performed by Image J software.

### 2.6. Protein Aggregation Assay and Proteasome Activity Assay

For aggresome detection, denuded oocytes were processed using the ProteoStat Aggresome Detection Kit (ENZ-51035-K10, Enzo Life Sciences, New York, NY, USA) with modifications. After IVM, COCs were treated with 0.1% hyaluronidase and vortexed for 30 s, washed in PBS-PVA, then fixed in 2% methanol-free formaldehyde in assay buffer for 1 h at room temperature. After permeabilization with 0.5% Triton X-100 in EDTA-supplemented buffer (3 mM, pH 8.0) for 1 h on ice, samples were stained with ProteoStat aggresome dye (1:2000 dilution) and Hoechst 33342 in BSA-containing buffer for 1 h with agitation. MG132 (10 μM), a proteasomal inhibitor, was included during the final 6 h of IVM as a positive control [33].

Proteasome activity was measured by incubating denuded oocytes with 5 μM Proteasome Activity Probe (I-190, R&D Systems, Minneapolis, MN, USA) for 1 h at 38.5 °C following three washes in IVM medium. Fluorescence signals were captured using fluorescence microscopy (Nikon, Tokyo, Japan). Analysis was performed by Image J software.

### 2.7. Immunofluorescence

After IVM, oocytes were washed in PBS-PVA, fixed in 4% PFA for 30 min at room temperature, and permeabilized with 0.5% Triton X-100/0.5% BSA in PBS for 15 min. After blocking with 1% BSA-PBS for 1 h, oocytes were incubated overnight at 4 °C with primary antibodies against GRP78 (11587-1-AP; Proteintech, Wuhan, China), and ATF6 (24169-1-AP; Proteintech, Wuhan, China). Oocytes were washed in PBS-PVA and then incubated with the corresponding secondary antibody for 1 hour at room temperature. Fluorescence signals were captured using fluorescence microscopy (Nikon, Tokyo, Japan). Analysis was performed by Image J software.

### 2.8. Real-Time Quantitative Polymerase Chain Reaction (RT-qPCR)

cDNA was synthesized with the Single Cell Sequence Specific Amplification Kit (P621-01, Vazyme, Jiangsu, China) according to the manufacturer’s protocol. RT-qPCR was performed using Taq Pro Universal SYBR qPCR Master Mix (Q712, Vazyme, Jiangsu, China) in a Bio-Rad CFX96 Touch™ system (Bio-Rad Laboratories, Inc. Hercules, CA, USA). Relative gene expression was calculated using the 2-ΔΔCt method with β-actin as endogenous control. The detailed information for primers was displayed in Table S1.

### 2.9. Western Blot

Western blot analysis of oocytes or cumulus cells was performed using standard procedures. Briefly, oocytes or cumulus cells were collected and boiled in sodium dodecyl sulfate (SDS) sample buffer for 5 min. The boiled proteins were separated by SDS-PAGE and then electrically transferred to PVDF membranes. The blots were probed with respective primary antibodies at an appropriate dilution by overnight incubation at 4 °C, followed by 1 h incubation with appropriate HRP-conjugated secondary antibodies at room temperature.

### 2.10. Smart-Seq2 and Data Preparation

The oocytes, CCs, and MGCs that were isolated from follicles were analyzed by RNA-Seq as previously described [34]. Briefly, the oocytes, CCs, and MGCs were rapidly transferred into the lysis buffer using a mouth pipette to ensure RNA integrity. Then we performed reverse transcription on the cell lysate and terminal deoxynucleotidyl transferase was adopted to add a poly A tail to the 3’ end of the first-strand cDNAs. Next, we performed 10–12 cycles of PCR to amplify the cDNA library. Final RNA-seq libraries were constructed using the TruePrep DNA Library Prep Kit V2 for Illumina (TD503-02, Vazyme, Jiangsu, China) according to the manufacturer’s instructions.

### 2.11. Sequencing Data Analysis

RNA-seq Smart-seq2 libraries were sequenced on Illumina NovaseqTM 6000 (Illumina, San Diego, CA, USA) [35]. Reads were first performed QC on the raw reads using cutadap (v2.10), then were aligned to the sheep genome (https://ftp.ensembl.org/pub/current_fasta/ovis_aries/dna_index/Ovis_aries.ARS-UI_Ramb_v3.0.dna.toplevel.fa.gz (accessed on 3 November 2025)) using HISAT2 software (version 2.2.1). The counting of fragments aligning per gene was performed using the featurecounts function of the Subread package (v1.4.6-p5). Seurat v4.0.3 (R v4.1.0) filtered low-quality cells (nCount_RNA ≥ 20,000; nFeature_RNA ≥ 1000; percent mitochondrial < 30%). Principal Component Analysis (PCA) dimensionality reduction was performed using the variable gene sets. Cell clusters were determined using a Shared Nearest Neighbor (SNN) modularity optimization-based clustering algorithm of the Seurat “FindClusters” function and were visualized with t-distributed Stochastic Neighbor Embedding (tSNE). The marker genes in tSNE plot were plotted by the FeaturePlot function in Seurat package (v4.0.3) [36].

### 2.12. Data Collection and Workflow of RNA-Seq Data Processing

We collected transcriptome data of *in vivo* and *in vitro* matured oocytes in different species, including human, human, monkey, and pig. All RNA sequencing (RNA-seq) data was derived from public databases, including gene expression omnibus (GEO) and Sequence Read Archive (SRA) with the following accession numbers: human (GSE158539), mouse (GSE165546), monkey (GSE233232), and pig (PRJNA237914).

In order to ensure the accuracy of data analysis, we processed raw sequencing data rather than preprocessed files. Transcriptomic datasets from different species were processed independently. First, FastQC (v0.11.8) was used to evaluate the quality of the raw RNA-seq data, and based on the quality control report, unqualified datasets were excluded. Next, Fastp (v0.23.1) was employed for further quality control to remove low-quality and unqualified reads. As a key sequence alignment tool, STAR (v2.7.0f) was used to align sequences to the respective reference genomes. The reference genomes for each species were as follows: Homo sapiens (assembly GRCh38.110), Mus musculus (assembly GRCm39.110), Macaca fascicularis (assembly Macaca_fascicularis_6.0), and Sus scrofa (assembly Sscrofa11.1). Finally, gene counts were generated using FeatureCounts (v1.6.3).

### 2.13. Differential Expression and Functional Enrichment

Differentially expressed genes (DEGs) were identified using the R package DESeq2 (v1.32.0) (|log2fold change| ≥ 1 and *p* < 0.05). Gene Ontology (GO) and Kyoto Encyclopedia of Genes and Genomes (KEGG) analysis were performed using DAVID (https://davidbioinformatics.nih.gov/ (accessed on 3 November 2025)) [37].

### 2.14. Cell–Cell Communication Analysis

To identify interactions between oocytes and their surrounding CCs, we used CellPhoneDB version 2 (https://github.com/Teichlab/cellphonedb (accessed on 3 November 2025)) to identify biologically relevant ligand–receptor interacting pairs from RNA-seq data, as described in previous study [29].

### 2.15. Statistical Analysis

Data were analyzed with SPSS 20.0. Gene expression, fluorescence intensities and developmental rates were subjected to t test or one-way ANOVA followed by Fisher’s LSD post hoc test. All experiments were performed with at least three biological replicates, and all values are presented as mean ± standard error (SEM). *p* value < 0.05 was considered statistically significant.

## 3. Results

### 3.1. Global Transcriptomic Landscape of Sheep Oocytes and Granulosa Cells Matured In Vivo and In Vitro

**Figure 1:**
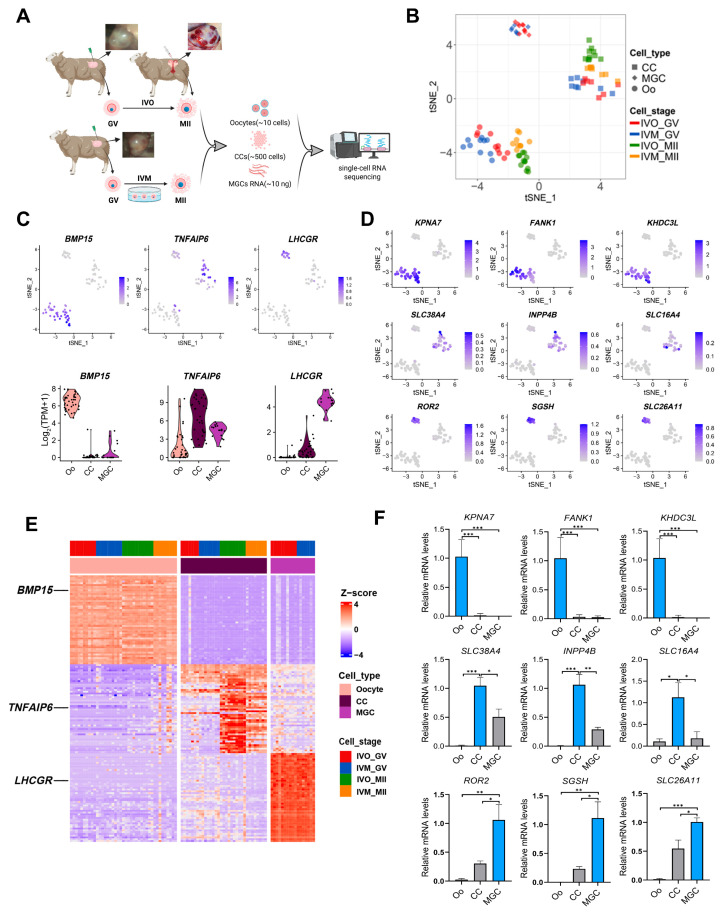
Global transcriptomes profiling of sheep oocytes and granulosa cells from *in vivo* and *in vitro* matured oocytes. (**A**) A schematic illustration of the study workflow. (**B**) The tSNE plots of oocytes and granulosa cells from the study, indicated by colors for different maturation stages and sources. Circles indicate oocytes (Oo), squares indicate cumulus cells (CC), and diamonds represent mural granulosa cells (MGC). (**C**) Expression patterns of oocyte, CC, and MGC marker genes exhibited on tSNE plots and Violin plots; a gradient of gray to blue indicates the low to high gene expression level. (**D**) Heatmap showing expression signatures of top 50 specifically expressed genes in each cell type; the value for each gene is row-scaled Z score. (**E**) Expression of the candidate cell-type-specific marker genes of oocyte, CC and MGC on tSNE plots. (**F**) RT-qPCR validation of candidate cell-type-specific marker genes of oocyte, CC and MGC. Statistical significance: * *p* < 0.05, ** *p* < 0.01, *** *p* < 0.001.

**Figure 2:**
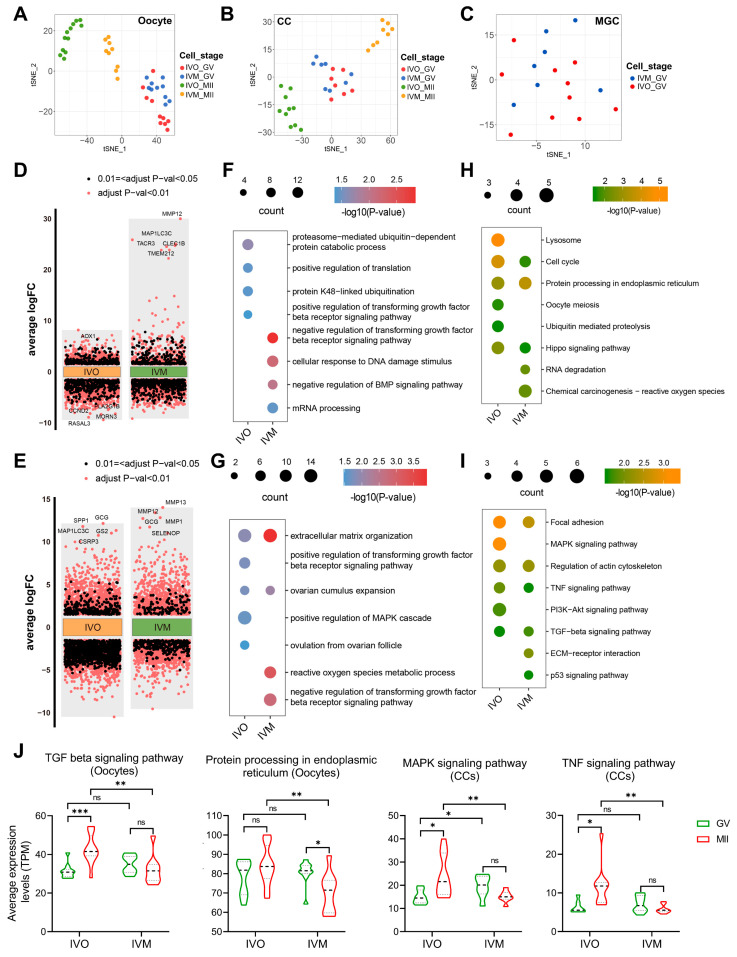
Transcriptomic analysis of GV and MII stage oocytes and cumulus cells from *in vivo* and *in vitro* matured oocytes. (**A**–**C**) The tSNE plots of oocyte, CC, and MGC, indicated by colors for different maturation stages and sources. (**D**,**E**) Differential gene expression analysis shows up- and down-regulated genes in oocytes and cumulus cells from *in vivo* and *in vitro* matured oocytes. An adjusted *p*-value < 0.01 is indicated in red, while an adjusted *p*-value < 0.05 (but ≥0.01) is indicated in black. (**F**–**I**) The GO (biological process) terms and KEGG pathways of up-DEGs between immature (GV) and mature (MII) stages in oocytes and cumulus cells. (**J**) Violin plots showing average expression levels of representative KEGG pathway-associated genes in oocytes or cumulus cells. Statistical significance: * *p* < 0.05, ** *p* < 0.01, *** *p* < 0.001.

To systematically dissect the global transcriptional signatures between physiologically (*in vivo*) and artificially (*in vitro*) matured ovine oocytes, we performed high-resolution RNA-seq on paired oocytes and their surrounding granulosa cell subpopulations, cumulus cells (CCs) and mural granulosa cells (MGCs) collected at both pre- and post-maturation stages (Figure 1A and Figure S1A). After filtering, a total of 41 oocyte samples, 31 CCs samples, and 17 MGCs samples were retained for subsequent analyses, with biological replicates showing high reproducibility (Figure S1B). Unsupervised t-distributed stochastic neighbor embedding (t-SNE) analysis revealed distinct clustering patterns between post-maturation oocytes and CCs according to their culture environments, while all pre-maturation cell types (oocytes, CCs, and MGCs) remained unsegregated in the t-SNE space (Figure 1B and Figure 2A–C). These data underscore that the *in vivo* versus *in vitro* environments significantly reshape the transcriptomic landscapes of both oocytes and their supporting granulosa cells.

We next annotated cell identities by mapping the canonical cell-type-specific marker genes for oocytes (BMP15), CCs (TNFAIP6) and MGCs (LHCGR) in the t-SNE embeddings [38,39,40,41] and validated their expression distributions with violin plots (Figure 1C). These markers, together with other established signatures, ranked among the top 50 differentially expressed genes (DEGs) in each cell respective type (Figure 1D). Besides these canonical identifiers, mining the top 50 DEGs revealed additional candidates with highly restricted expression patterns: oocyte-enriched KPNA7, SLC38A4 and ROR2, CC-specific FANK1, INPP4B and SGSH, and MGC-restricted KHDC3L, SLC16A4 and SLC26A11 (Figure 1E and Figure S1C). Independent RT-qPCR confirmed the cell-type-restricted expression of each candidate marker, fully corroborating the transcriptomic data (Figure 1F). These newly identified marker genes provide a foundation for the future identification and characterization of discrete ovine follicular cell populations.

To dissect the cell type-specific transcriptional reprogramming from the germinal vesicle (GV) to metaphase II (MII) stage under *in vivo* (IVO)and *in vitro* maturation (IVM) conditions, we independently identified the DEGs between GV and MII in oocytes and CCs (Figure 2D,E). Gene Ontology and KEGG enrichment analyses of oocyte up-regulated DEGs revealed starkly divergent biological themes. *In vivo* matured oocytes were significantly enriched for translational regulation, protein processing, and the ubiquitin-proteasome system (UPS) (Figure 2F,H), a pivotal pathway for clearance of misfolded proteins [42]. In contrast, *in vitro* matured oocytes displayed predominant activation of mRNA processing and DNA damage response pathways. Consistently, cumulus cells matured *in vitro* exhibited robust upregulation of reactive oxygen species (ROS) metabolism, likely underpinning the heightened DNA damage signature in companion oocytes (Figure 2G,I). Moreover, the TGFβ receptor signaling pathway, which is critical for oocyte maturation [43,44], was selectively potentiated in the *in vivo* maturation environment in both oocytes and cumulus cells, whereas its activity was suppressed under *in vitro* conditions. The pathway-associated genes related to protein homeostasis and autocrine/paracrine signaling exhibited divergent dynamics: their mean expression progressively intensified during *in vivo* maturation yet declined steadily under *in vitro* conditions (Figure 2J). This inverse regulation likely contributes to the functional disparities in oocyte quality observed between the two maturation systems.

### 3.2. Conserved Determinants of Oocyte Maturation: A Cross-Species Comparison Between In Vivo and In Vitro Environments

**Figure 3:**
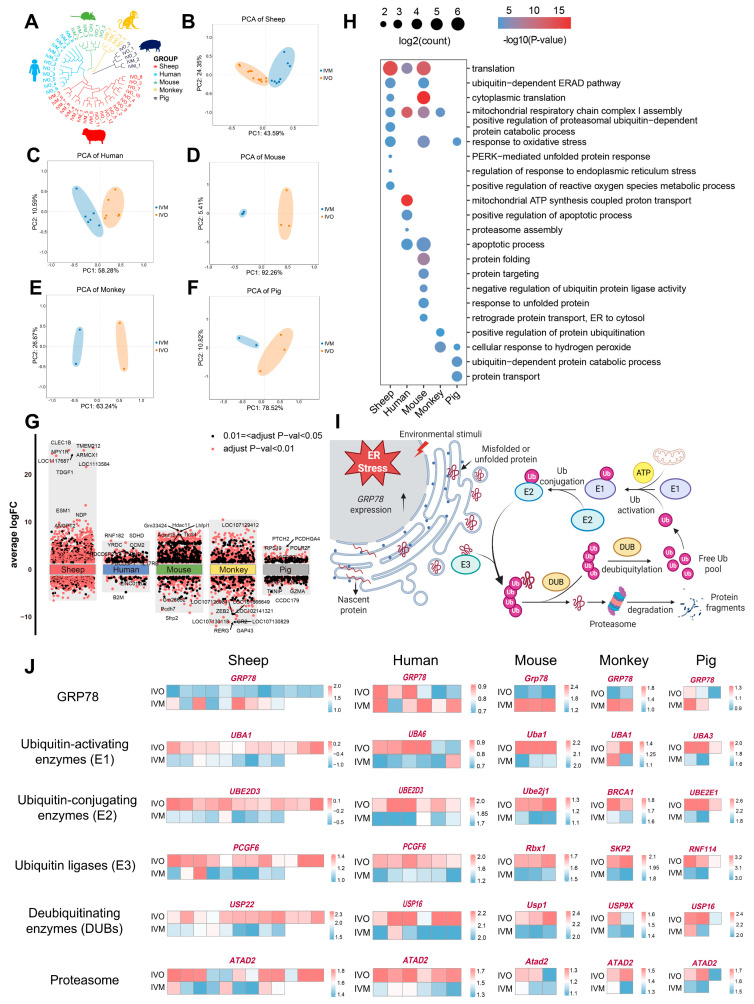
Conserved features of oocyte maturation: comparing *in vivo* and *in vitro* environments across species. (**A**) Hierarchical clustering of the transcriptome landscape of oocyte from *in vivo* and *in vitro* matured oocytes across different species; different colors represent different species: red, sheep; blue, human; green, mouse; yellow, monkey; and purple, pig. (**B**–**F**) Principal component analysis (PCA) comparing *in vivo*- and *in vitro* matured oocytes across multiple species. (**G**) Differential gene expression analysis shows up- and down-regulated genes in oocytes from *in vivo* and *in vitro* matured oocytes across different species. An adjusted *p*-value < 0.01 is indicated in red, while an adjusted *p*-value < 0.05 (but ≥0.01) is indicated in black. (**H**) GO enrichment of differential genes in oocytes from *in vivo* and *in vitro* matured oocytes across different species. (**I**) Transcriptomic analysis reveals ubiquitin-mediated degradation of protein aggregates in response to stimuli. (Created in BioRender. Cui, J. (2025) https://BioRender.com/0d7d9kl accessed on 7 December 2025). (**J**) Heatmaps showing scaled expression levels of endoplasmic reticulum (ER) stress marker GRP78 and ubiquitin-proteasome pathway components (E1, E2, E3, DUBs, proteasome) across multiple species.

To interrogate the evolutionary conservation of oocyte maturation programs under *in vivo* versus *in vitro* conditions, we retrieved publicly available RNA-seq datasets of *in vivo* and *in vitro* matured oocytes from human, mouse, monkey, and pig [45,46,47,48] and integrated them with our ovine IVO and IVM transcriptomes for cross-species comparison. As expected, the unsupervised clustering revealed pronounced species-specific transcriptomic signatures (Figure 3A). Within each species, IVO and IVM oocytes formed discrete clusters, underscoring the dominant influence of the maturation environment (Figure 3B–F).

We next conducted DGE analyses between *in vivo* and *in vitro* matured oocytes across all five species, followed by functional annotation and pathway enrichment of the resulting DEGs (Figure 3G,H and Figure S2A). Despite species divergence, core biological processes and pathways were recurrently enriched, including “translation”, “response to oxidative stress” and “ubiquitin-dependent ER-associated degradation (ERAD)”. Notably, species-specific modules were also identified and converged on protein quality control mechanisms, particularly in endoplasmic reticulum protein processing and UPS-mediated clearance of misfolded proteins. Oocyte maturation demands ER homeostasis to sustain high-level protein synthesis; however, oxidative stress during IVM perturbs ER function, resulting in misfolded protein accumulation [49]. Consistently, our enrichment analyses indicate that IVM conditions compromise ER proteostasis across multiple mammalian species, thereby providing a conserved mechanistic explanation for the suboptimal developmental competence of IVM oocytes.

To validate this hypothesis, we quantified ER stress marker GRP78 and key UPS constituents, including E1 ubiquitin-activating enzymes, E2 ubiquitin-conjugating enzymes, E3 ubiquitin ligases, deubiquitinating enzymes (DUBs), and proteasome subunits across IVO and IVM oocytes from all five species. In every species examined, IVM oocytes displayed markedly elevated GRP78 transcript levels accompanied by a coordinated down-regulation of UPS genes (Figure 3I,J and Figure S2B–E). These findings indicate that IVM milieu universally induces ER stress and attenuates protein quality control capacity, leading to misfolded protein accumulation and compromised developmental competence.

### 3.3. Ligand–Receptor Interactions Between Oocytes and Cumulus Cells in In Vivo and In Vitro Maturation Environments

**Figure 4:**
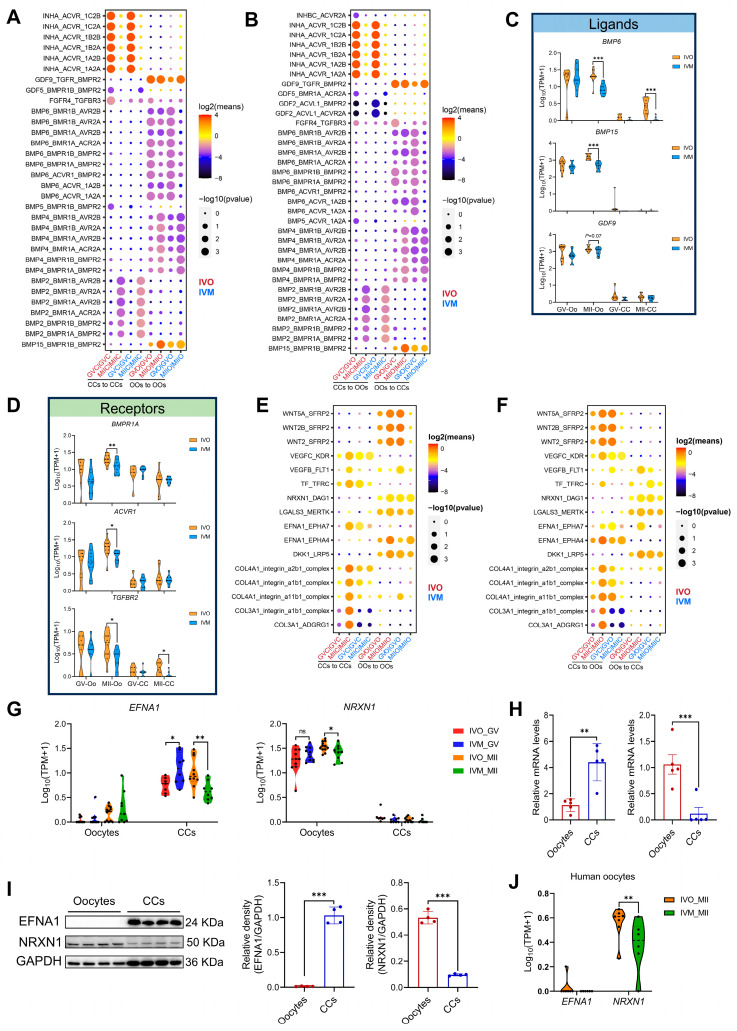
Ligand–receptor interactions between oocytes and cumulus cells in different maturation environments. (**A**,**B**) CellPhoneDB analysis of TGF-β/BMP signaling interactions between oocytes and cumulus cells (autocrine and paracrine) in different maturation environments. GVC, GV stage-cumulus cell; MIIC, MII stage-cumulus cells; GVO, GV oocyte; MIIO, MII oocyte. (**C**,**D**) Violin plots of selected TGF-β/BMP signaling ligand and receptor genes in oocytes and cumulus cells. (**E**,**F**) Distinct ligand–receptor interactions between oocytes and cumulus cells (autocrine and paracrine) in *in vivo* versus *in vitro* maturation (IVM) environments. (**G**) EFNA1 and NRXN1 expression was shown in violin plots. (**H**,**I**) Expression of EFNA1 and NRXN1 mRNA and protein expression levels in oocytes and cumulus cells. (**J**) Expression of EFNA1 and NRXN1 in human oocytes was visualized using violin plots. Statistical significance: * *p* < 0.05, ** *p* < 0.01, *** *p* < 0.001.

Autocrine and paracrine signaling between oocytes and their surrounding CCs is a well-established determinant of both meiotic progression and subsequent embryogenesis [11]. Notably, our transcriptomic data revealed a pronounced suppression of both TGF-β and BMP signaling in oocytes and CCs under *in vitro* maturation conditions, in stark contrast to their robust activation *in vivo* (Figure 2F,G). To comprehensively delineate how the maturation environment modulates autocrine and paracrine signaling between oocytes and CCs, we employed CellPhoneDB v2.0 to systematically interrogate ligand–receptor interactions across these two cell types. First, we mapped TGF-β/BMP pathway pairs across maturation environments. Strikingly, *in vitro* culture markedly weakened the interactions GDF9-TGFBR-BMPR2 and BMP15-BMPR1B-BMPR2 (Figure 4A,B), which are known to prevent granulosa cell apoptosis and promote oocyte developmental competence [50,51]. Concomitantly, transcript levels of BMP receptor type II (BMPR2) and its cognate ligands BMP6, BMP15 and GDF9 were significantly reduced in *in vitro* matured oocytes (Figure 4C,D). Extending this analysis to WNT signaling, another critical pathway for oocyte maturation, we observed analogous attenuation of WNT5A-SFRP4, DKK1-LRP6, and RSPO1-LRP4 interactions under *in vitro* conditions (Figure S3A–D).

We next systematically screened for ligand–receptor pairs whose autocrine/paracrine signaling strength was consistently higher *in vivo* than *in vitro* (Figure 4E,F). Stringent screening criteria yielded 16 high-confidence ligand–receptor pairs comprising 12 distinct ligands. Notably, 10 of these ligands have been previously demonstrated, or implicated via homologs, to enhance oocyte maturation and blastocyst formation. For instance, WNT5A, VEGFA, and CXCL12 activate MAPK and modulate canonical WNT signaling to promote porcine oocyte maturation; exogenous transferrin enhances oocyte IVM efficiency and blastocyst rates; and LGALS governs meiotic resumption and fertilization competence [52,53,54,55]. These concordances collectively corroborate the reliability of our screening methodology. Strikingly, EFNA1 and NRXN1 emerged as uncharacterized regulators of oocyte development. We found that EFNA1 is primarily secreted by CCs, whereas NRXN1 originates from oocytes (Figure 4G–I). Both ligands and their cognate receptors exhibited markedly lower expression under *in vitro* conditions (Figure S3E–G), implying weakened oocyte–CCs signaling. Notably, consistent with observations in ovine oocytes, human oocytes display elevated NRXN1 expression, with significantly higher levels detected in *in vivo* matured oocytes compared to those matured *in vitro*, whereas EFNA1 is virtually undetectable in oocytes (Figure 4J). Functional interrogation of EFNA1 and NRXN1 may therefore uncover new targets for improving IVM protocols.

### 3.4. EFNA1 and NRXN1 Enhance Oocyte Developmental Competence

**Figure 5:**
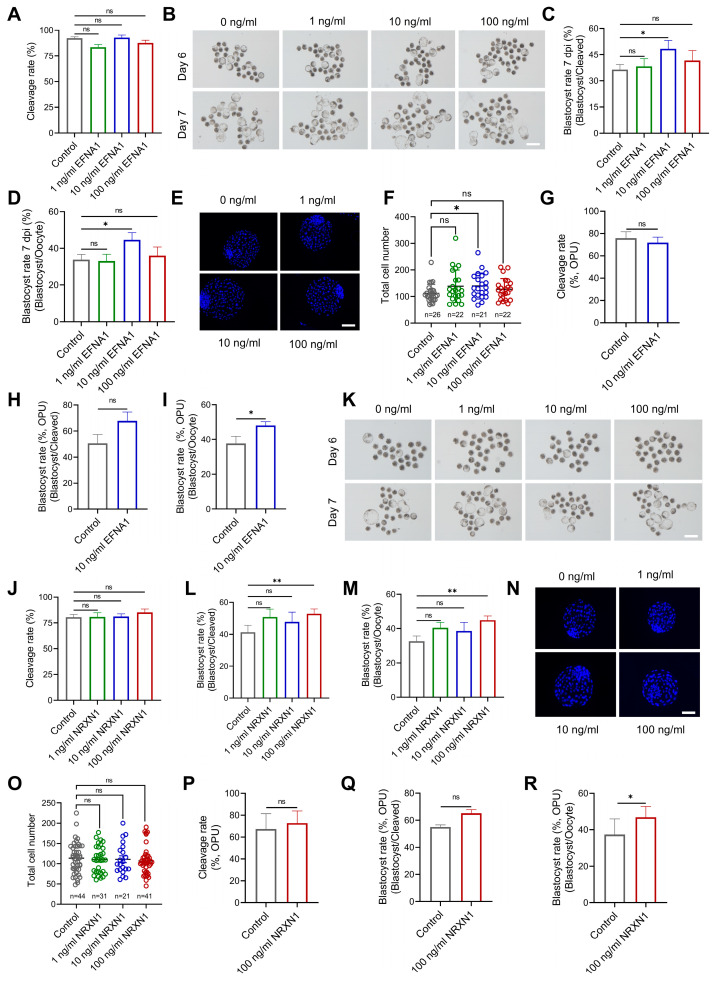
Supplementation of EFNA1 and NRXN1 in IVM medium improves oocyte developmental competence. (**A**) Effects of EFNA1 supplementation during IVM on cleavage rate in abattoir-derived oocytes. (**B**) Representative images of day 6 and day 7 blastocysts in control and EFNA1-treated groups (Scale bar = 400 µm). (**C**,**D**) Effects of EFNA1 supplementation during IVM on blastocyst formation rate (per oocyte and per cleaved embryo) in abattoir-derived oocytes. (**E**,**F**) Representative images of blastocysts showing total cell number and quantification of total cell numbers (Scale bar = 100 µm). (**G**–**I**) Effects of NRXN1 supplementation during IVM on cleavage rate, blastocyst formation rate (per oocyte and per cleaved embryo) in LOPU-derived oocytes. (**J**) Effects of NRXN1 supplementation during IVM on cleavage rate in abattoir-derived oocytes. (**K**) Representative images of day 6 and day 7 blastocysts in control and NRXN1-treated groups (Scale bar = 400 µm). (**L**,**M**) Effects of NRXN1 supplementation during IVM on blastocyst formation rate (per oocyte and per cleaved embryo) in abattoir-derived oocytes. (**N**,**O**) Representative images of blastocysts showing total cell number and quantification of total cell numbers (Scale bar = 100 µm). (**P**–**R**) Effects of NRXN1 supplementation during IVM on cleavage rate, blastocyst formation rate (per oocyte and per cleaved embryo) in LOPU-derived oocytes. Statistical significance: * *p* < 0.05, ** *p* < 0.01.

To investigate the functional impact of EFNA1 and NRXN1 on ovine oocyte maturation, each cytokine was individually added to a chemically defined IVM medium. Slaughterhouse-derived oocytes were matured for 24 h, fertilized *in vitro*, and cultured for 7 days. Individual treatment with EFNA1 (10 ng/mL) or NRXN1 (100 ng/mL) significantly improved oocyte developmental competence, as evidenced by elevated blastocyst yields relative to both initial oocyte numbers and cleaved embryos (Figure 5A–D, J–M). EFNA1 supplementation markedly increased total blastocyst cell numbers, indicating a pronounced enhancement in embryo quality, while NRXN1 did not alter blastocyst cellularity (Figure 5E,F,N,O).

To extend these findings to a clinically relevant setting, we applied the same supplementation strategy to oocytes obtained by laparoscopic ovum pick-up (LOPU) followed by IVM. Post-IVF development to blastocyst was again significantly improved by either EFNA1 or NRXN1, with no effect on the progression from cleaved embryo to blastocyst (Figure 5H,Q). Additionally, both ligands increased the proportion of oocytes reaching metaphase II (Figure S4A–C). Collectively, our results identify EFNA1 and NRXN1 as pivotal autocrine/paracrine signaling ligands that markedly elevate the developmental quality of *in vitro* matured oocytes.

### 3.5. EFNA1 Alleviates Excessive Misfolded Protein Accumulation in Oocytes During IVM

**Figure 6:**
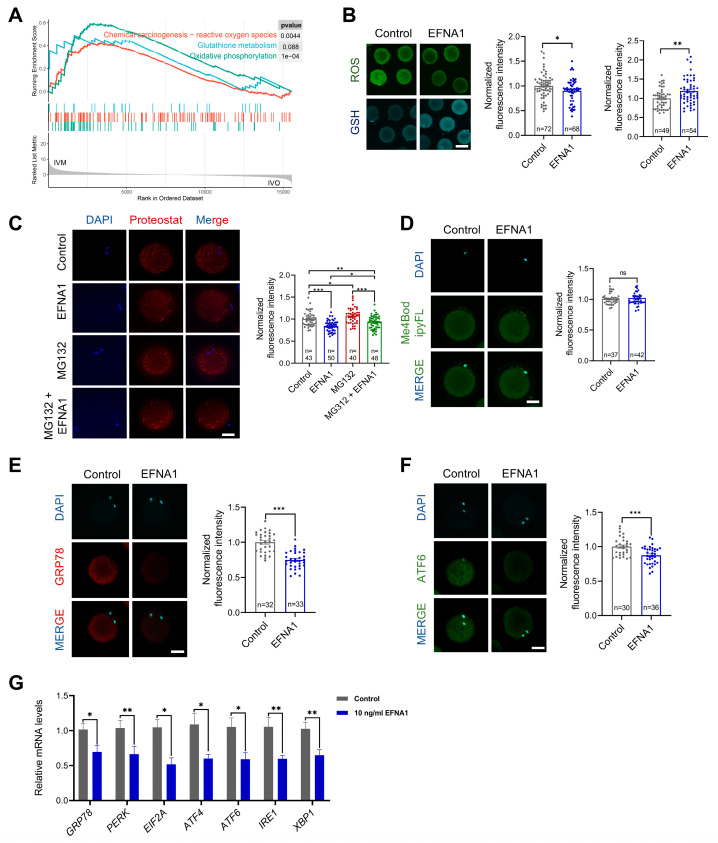
EFNA1 alleviates excessive misfolded protein accumulation in oocytes during IVM. (**A**) GSEA analysis of differential expression genes related to oxidative phosphorylation, glutathione metabolism, and reactive oxygen species in *in vitro* matured oocytes compared to *in vivo*. (**B**) Effect of 10 ng/mL EFNA1 on ROS and GSH levels in matured sheep oocytes. Intracellular ROS and GSH levels were measured using CM-H2DCFDA and CellTracker™ Blue CMF2HC fluorescent probes, respectively (Scale bar = 200 µm). (**C**) Effect of 10 ng/mL EFNA1 on protein aggregates content in matured sheep oocytes. The protein aggregates content was determined by proteoStat aggresome detection reagent labeling (Scale bar = 50 µm). (**D**) Effect of 10 ng/mL EFNA1 on proteasome activity in matured sheep oocytes. The proteasome activity was evaluated by Me4BodipyFL (Scale bar = 50 µm). (**E**,**F**) Immunofluorescence detection of ER stress-associated protein (GRP78, ATF6) in matured sheep oocytes cultured with or without EFNA1 (Scale bar = 50 µm). (**G**) Relative expression levels of ER stress-associated genes (GRP78, PERK, EIF2A, ATF4, ATF6, IRE1, XBP1) in matured sheep oocytes cultured with or without EFNA1 as analyzed by RT-PCR. Statistical significance: * *p* < 0.05, ** *p* < 0.01, *** *p* < 0.001.

Excessive ROS disrupts redox-dependent protein folding in the ER, provoking misfolded proteins accumulation and ER stress in oocytes [49,56]. Compared to their *in vivo* counterparts, *in vitro* matured oocytes display aberrant oxidative phosphorylation and marked ROS overproduction (Figure 6A), implicating oxidative imbalance as a primary driver of ER stress during IVM. EFNA4, a ligand of the same family, upregulates GPX4 and suppresses ferroptosis by curbing ROS [57]; we therefore postulated that EFNA1 exerts similar antioxidant functions. Supplementing the IVM medium with EFNA1 indeed fortified oocyte antioxidant defenses, evidenced by diminished ROS and elevated GSH (Figure 6B). EFNA1 also reduced misfolded protein aggregates and rescued the proteasome inhibitor MG132-induced aggregation phenotype (Figure 6C), yet left proteasome activity unchanged (Figure 6D). Immunofluorescence and qPCR revealed significant down-regulation of the ER stress markers GRP78 and ATF6, alongside attenuated expression of the full UPR gene set GRP78, PERK, EIF2A, ATF4, ATF6, IRE1 and XBP1 (Figure 6E–G).

### 3.6. NRXN1 Mitigates Aberrant Lipid Deposition and ER Stress in Oocytes During IVM

**Figure 7:**
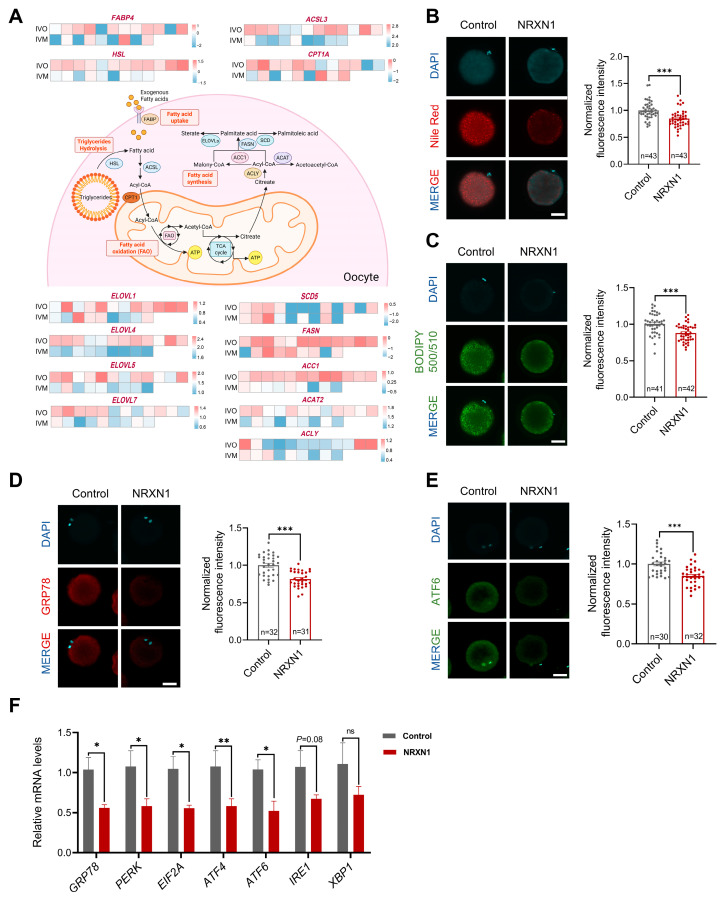
NRXN1 mitigates aberrant lipid deposition and ER stress in oocytes during IVM. (**A**) Heatmaps showing scaled expression levels of lipid metabolism genes in oocytes from *in vivo* and *in vitro* matured oocytes. (Created in BioRender. Cui, J. (2025) https://BioRender.com/onf1fa2 accessed on 7 December 2025). (**B**) Effect of 100 ng/mL NRXN1 on lipid droplets content in matured sheep oocytes. Intracellular lipid droplets content was measured using Nile-red (Scale bar = 50 µm). (**C**) Effect of 100 ng/mL NRXN1 on fatty acid contents in matured sheep oocytes. The lipid content was determined by BODIPY 500/510 labeling (Scale bar = 50 µm). (**D**,**E**) Immunofluorescence detection of ER stress-associated protein (GRP78, ATF6) in oocytes cultured with or without NRXN1 (Scale bar = 50 µm). (**F**) Relative expression levels of ER stress-associated genes (*GRP78*, *PERK*, *EIF2A*, *ATF4*, *ATF6*, *IRE1*, *XBP1*) in oocytes cultured with or without NRXN1 as analyzed by RT-PCR. Statistical significance: * *p* < 0.05, ** *p* < 0.01, *** *p* < 0.001.

Aberrant lipid metabolism disrupts endoplasmic reticulum homeostasis [17,58]. Previous studies have demonstrated that bovine oocytes matured *in vitro* exhibit excessive lipid accumulation, which is not observed in their *in vivo* counterparts, resulting in impaired developmental competence [16]. To determine whether a similar metabolic dysregulation occurs in sheep, we quantified expression of genes governing fatty acid uptake (FABP), triglycerides hydrolysis (HSL), fatty acid β-oxidation (ACSL3, CPT1A), and de novo lipogenesis (ELOVLs, SCD5, FASN, ACC1, ACAT2, ACLY). As in bovine, IVM ovine oocytes also displayed marked dysregulation of these lipid metabolic genes (Figure 7A). Given NRXN1’s reported capacity to modulate lipid metabolism and lipid storage [59], we postulated that its beneficial effects on oocyte competence are mediated by limiting aberrant lipid accretion. Indeed, supplementation of IVM medium with NRXN1 substantially decreased lipid droplet abundance and total fatty acid content (Figure 7B,C). Concomitantly, NRXN1 significantly downregulated key ER stress markers, including GRP78 and ATF6 at the protein level and the UPR genes (GRP78, PERK, EIF2A, ATF4, ATF) at the transcript level (Figure 7D–F). Collectively, these findings indicate that NRXN1 attenuates ER stress in IVM oocytes, at least in part, by preventing lipid overload.

### 3.7. Synergistic EFNA1-NRXN1 Supplementation in IVM Medium Improves Oocyte Developmental Competence and Blastocyst Quality

**Figure 8:**
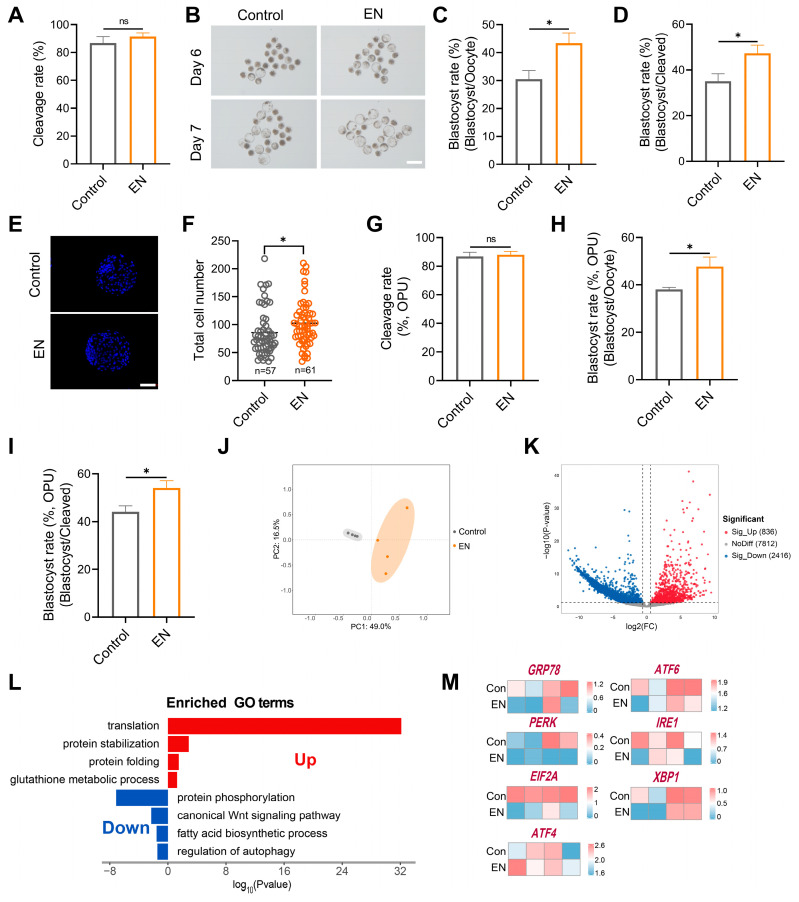
Combined EFNA1 and NRXN1 supplementation enhances oocyte developmental competence and rewires transcriptional landscape. (**A**) Effects of EFNA1 combined with NRXN1 (EN) supplementation during IVM on cleavage rate in abattoir-derived oocytes. (**B**) Representative images of day 6 and day 7 blastocysts in control and EN-treated groups (Scale bar = 400 µm). (**C**,**D**) Effects of EN supplementation during IVM blastocyst formation rate (per oocyte and per cleaved embryo) in abattoir-derived oocytes. (**E**,**F**) Representative images of blastocysts showing total cell number and quantification of total cell numbers (Scale bar = 100 µm). (**G**–**I**) Effects of EN supplementation during IVM on cleavage rate, blastocyst formation rate (per oocyte and per cleaved embryo) in LOPU-derived oocytes. Different letters denote significant differences, ns *p* > 0.05, * *p* < 0.05. (**J**) PCA showing the reproducibility between repetitive samples and the difference between groups. (**K**) DEGs between control and EN oocytes (co-treated with EFNA1 and NRXN1) were shown in the volcano map. Genes that expressed higher (up-regulated) in EN oocytes are shown in red, and genes that were lower expressed (down-regulated) are shown in blue. (**L**) GO enrichment of up-regulated DEGs and down-regulated DEGs. Red represents upregulation and blue represents downregulation. (**M**) Heatmaps showing scaled expression levels of ER stress-associated genes (*GRP78*, *PERK*, *EIF2A*, *ATF4*, *ATF6*, *IRE1*, *XBP1*) in oocytes from control and EN-treated groups. Statistical significance: * *p* < 0.05.

To translate our findings into commercial scale embryo production, we combined the optimal concentrations of EFNA1 (10 ng/mL) and NRXN1 (100 ng/mL) into a single supplement (EN) and incorporated it into standard IVM medium. In both abattoir-derived and LOPU-IVM systems, EN co-treatment synergistically elevated blastocyst yields (total blastocysts per oocyte) relative to untreated controls and single-factor supplementation (Figure 8A–D). Whereas NRXN1 alone did not augment Day-7 blastocyst cell number, the EN combination significantly increased total cellularity (Figure 8E,F). Critically, EN not only advanced oocyte competence but also ensured seamless progression from cleavage to blastocyst in the clinically relevant LOPU-IVM workflow (Figure 8G–I), thereby achieving a level of efficiency unattainable with individual factors.

To define the global transcriptional consequences of EN exposure, we subjected EN-treated and control oocytes to scRNA-seq. Principal component analysis (PCA) revealed clear segregation of transcriptomic patterns between EN-treated and untreated oocytes (Figure 8J). Gene Ontology enrichment indicated that EN co-treatment simultaneously strengthened antioxidant defenses, preserved ER redox homeostasis for accurate protein folding, curtailed de novo lipogenesis, and downregulated the ER-stress gene set (Figure 8L,M). The dual-factor regimen thus retains the distinct biological actions of each ligand while eliciting synergistic effects.

## 4. Discussion

*In vitro* oocyte maturation serves as one of the important techniques in both human assisted reproduction medicine and animal breeding, yet broader adoption is hindered by the persistent suboptimal quality of *in vitro* matured mammalian oocytes. Our study reveals that the maturation environment profoundly reshapes the ovine oocyte transcriptome, with cross-species analysis confirming disrupted ER homeostasis as a conserved feature of *in vitro* maturation. By mapping ligand–receptor interactions between oocytes and cumulus cells across maturation conditions, we identified two pivotal regulators capable of restoring oocyte ER homeostasis impaired during *in vitro* maturation. Consequently, our study provides crucial insights into the suboptimal quality of *in vitro* matured oocytes. Importantly, our findings pave the way for refining IVM protocols through the therapeutic supplementation of the two identified factors, which enhance developmental competence by restoring ER homeostasis.

We found excessive ROS generation occurs in *in vitro* matured ovine oocytes, corroborating previous reports [12,60]. Excess ROS disrupted endoplasmic reticulum redox homeostasis, impaired protein folding and drove misfolded protein accumulation [49,56], a defect conserved across sheep, humans, mouse, monkey, and pig oocytes. IVM also induced abnormal lipid accumulation that was absent *in vivo* and further amplified ER stress [16]. ER stress, in turn, intensified ROS production and lipid dysregulation [58], establishing a self-perpetuating cycle that significantly compromised the oocyte quality [13,61]. Therefore, preventing excessive ROS generation and lipid accumulation may constitute an essential strategy to mitigate ER stress, preserve ER physiological function, and ultimately improve oocyte developmental competence during IVM.

Disrupted autocrine and paracrine signaling within the cumulus–oocyte complex is tightly linked to severe oocyte maturation defects [62,63]. Our transcriptomic analysis demonstrates markedly divergent ligand–receptor networks between *in vivo* and *in vitro* matured oocytes. We further identify EFNA1 and NRXN1 as novel endogenous regulators that mitigate ER stress and enhance oocyte developmental competence. Unlike the ER-stress inhibitor tauroursodeoxycholic acid (TUDCA), whose clinical use is limited by dose-dependent toxicity [64,65], EFNA1 and NRXN1 exhibit excellent tolerability while preserving oocyte quality, offering a safer option for IVM media. Their combined supplementation synergistically elevated both blastocyst yield (per oocyte) and, more critically, blastocyst conversion (per cleaved embryo) in the LOPU-IVM system, an outcome unattainable with either ligand alone. Our results align with accumulating evidence that multifactorial supplementation outperforms single-factor approaches: the combined use of follicular fluid-derived FGF2, LIF and IGF1 (FLI) markedly surpasses individual components in enhancing oocyte competence [66,67,68,69], and sequential treatment with C-type natriuretic peptide (CNP), melatonin (MT) and FLI yields superior oocyte quality compared with CNP alone or CNP + MT dual treatment [70]. Collectively, targeted multi-ligand cocktails represent a powerful strategy for advancing contemporary IVM technology.

ER stress, triggered by *in vitro* maturation conditions, is a critical factor impairing oocyte developmental competence [71]. Our study demonstrates that EFNA1 mitigates ER stress by enhancing cellular antioxidant defenses and reducing misfolded protein accumulation, whereas NRXN1 alleviates ER stress through lipid-homeostatic mechanisms that sustain ER integrity. Notably, EFNA1 has been identified as a novel EGFR ligand that drives epithelial–mesenchymal transition (EMT) in gastric cancer [72]. EGFR signaling is also indispensable for cumulus cell expansion, an essential prerequisite for oocyte maturation and ovulation [73]. Moreover, EFNA1 activates focal adhesion kinase-mediated cellular motility [74], and cumulus expansion is likewise FAK-dependent [75]. These convergent pathways suggest that EFNA1 may directly modulate cumulus cell function during oocyte maturation, a hypothesis that now warrants rigorous investigation. Although a previous study demonstrated that NRXN1 promotes neurite outgrowth through FGFR1 activation and FGFR1 governs lipid droplet dynamics [76,77,78], we observed no change in FGFR1 expression in NRXN1-treated oocytes. For this reason, we speculate that NRXN1 modulates lipid metabolism through alternative mechanisms, warranting further investigation to elucidate the underlying pathways.

## 5. Conclusions

**Figure 9:**
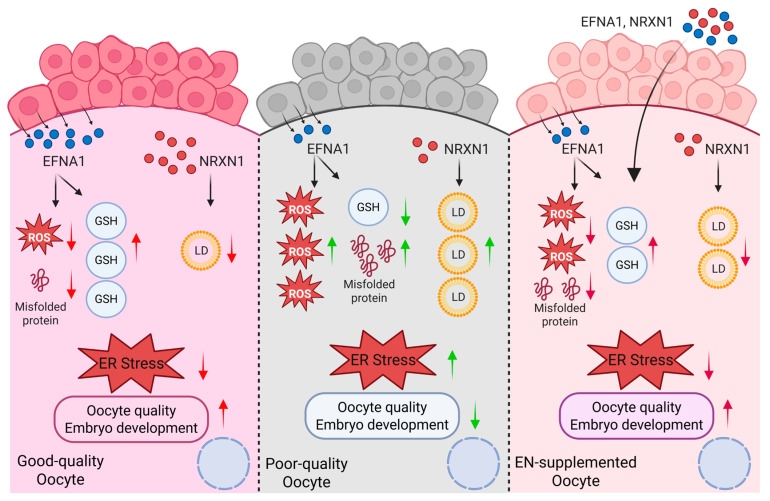
Schematic model illustrating EFNA1 and NRXN1 in promoting oocyte developmental competence (Created in BioRender. Cui, J. (2025) https://BioRender.com/o1mm3e0 accessed on 7 December 2025).

In conclusion, this study performed ligand–receptor mapping of oocyte–cumulus cell crosstalk to identify EFNA1 and NRXN1 as key factors absent in current IVM systems. Supplementation of either ligand alone in IVM medium significantly improved maturational quality and developmental competence in both abattoir-derived and LOPU-retrieved oocytes, acting via complementary mechanisms that attenuate ER stress (Figure 9). Importantly, their combined administration elicited a synergistic increase in blastocyst conversion within the LOPU-IVM system, underscoring their value for optimizing mammalian *in vitro* embryo production. Although ER stress during IVM is an evolutionarily conserved constraint across mammalian species, the translational potential of the EFNA1-NRXN1 combination for assisted reproductive technologies in other taxa remains to be established and is the focus of ongoing investigation.

## Data Availability

The original contributions presented in this study are included in the article/Supplementary Material. Further inquiries can be directed to the corresponding authors.

## Supplementary Materials

The following supporting information can be downloaded at https://www.mdpi.com/article/10.3390/antiox14121499/s1. Table S1 Oligonucleotide primer sequences used for quantitative real-time PCR in sheep; Figure S1: Quality control and expression of candidate cell type specific markers; Figure S2: Conserved features of oocyte maturation: comparing in vivo and in vitro environments across species; Figure S3: WNT signaling interactions between oocytes and cumulus cells in different maturation environments; Figure S4: Effects of EFNA1 and NRXN1 on oocyte nuclear maturation.
