# Supplementary material for: Cross-Species Insights into In Vitro Maturation Defects of the Oocyte and Identification of Crucial Regulators for Sheep Oocyte Maturation
Source: Antioxidants (Basel). 2025 Dec 13;14(12):1499. doi: 10.3390/antiox14121499 (PMC12730067; doi:10.3390/antiox14121499)
Supplement: Supplementary file 1 [file antioxidants-14-01499-s001.zip › Supplementary Material 1.pdf]

Supplementary Material 1

Table S1 Oligonucleotide primer sequences used for quantitative real-time PCR in sheep.

Oligonucleotide primer sequences used for quantitative real-time PCR in sheep.

Gene	Primer sequence (5' -3')	Reference/accession numbers	Product size (bp)
KPNA7	F: CCTCGGGAACATAGCAGGTG<br>R: AGAGGGGTACGGGTTCTTGT	XM_015104257.4	169
FANK1	F: GAAGAAAGCAAAGCGGCAGG<br>R: TCGCGTAGCCCGTATAAATGA	XM_060404584.1	107
KHDC3L	F: GGTTTCCGACGCTCGTTCAG<br>R: AATGAAACCAGTAGGGCCGC	XM_042254559.2	90
SLC38A4	F: ATGAGAGCATCAGTGGCGAG<br>R: AGGTGGTCCCAGGATGATGT	XM_042246822.2	183
INPP4B	F: GGTGAACCGAGCTAAGCAGT<br>R: TGCTTGGCTGAAGACGATGT	XM_042234317.1	112
SLC16A4	F: AGGTTGGGGATGGATGGTC<br>R: ACAGCAACTAGGGGACCGA	XM_042252148.2	124
ROR2	F: TCCACCTACAACAGTTCGGC<br>R: CATTGCTGACGTTGCTGACC	XM_027964188.2	94
SGSH	F: GGCATTCACACAGGCATCAT<br>R: ACAGAGCCATTCTCCTCCGT	XM_042256793.2	92
SLC26A11	F: CTGTCCATCCTGAGATGCCC<br>R: CCTGGAAGGAGTATGCGACC	XM_027974246.3	120
GRP78	F: AGCCCTATAGCTGCCTGCTG<br>R: CCACGTCCTCCTTCTTGTCC	XM_004005637.4	131
PERK	F: AGTGGAAAGGTGAGGTACATCTG<br>R: CAACGGCTCTCACGGTTTTC	XM_004005901.6	121
EIF2A	F: TCAGTTGGAGAAAATTCAGAAAGAG<br>R: TTAAATTCCCAGTTCCAAATCTTCC	XM_027959332.3	70
ATF4	F: AGACAACAGCACGGAGGATG<br>R: TCTGGCATGGTTTCCAGGTC	XM_012158819.3	120
ATF6	F: GCTCTCTCAGCCTACCGTGG<br>R: CACAGGGGCTGGTACCACAT	XM_042256929.1	130
IRE1A	F: CAACCACTCGCTCCACTCC<br>R: CCTCATCCTCGTCGTCCTG	XM_027974337.2	80
XBP1	F: GCTTTCAACCAGCAACTGCC<br>R: GGGTCCTCCCAAGAATGGTC	XM_004017459.5	135
β-actin	F: GTCACCAACTGGGACGACA<br>R: AGGCGTACAGGGACAGCA	NM_001009784.3	208
