# Supplementary material for: Cross-Species Insights into In Vitro Maturation Defects of the Oocyte and Identification of Crucial Regulators for Sheep Oocyte Maturation
Source: Antioxidants (Basel). 2025 Dec 13;14(12):1499. doi: 10.3390/antiox14121499 (PMC12730067; doi:10.3390/antiox14121499)
Supplement: Supplementary file 1 [file antioxidants-14-01499-s001.zip › Supplementary Material 2-5.pdf]

Supplementary Material 2-5

Figure S1

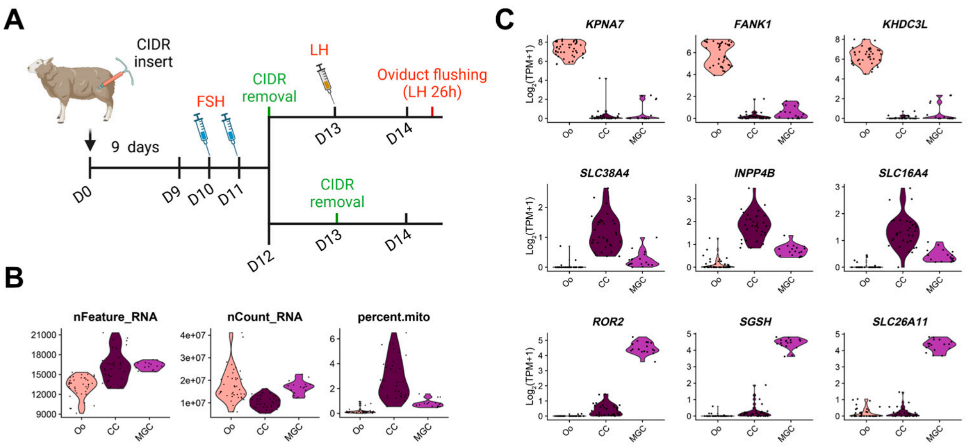

Supplementary Material 2: **Figure S1.** Quality control and expression of candidate cell type specific markers. (**A**) Hormonal superovulation treatment and sample collection timing of oocytes and granulosa cells in sheep. (**B**) Violin plots showing the number of features, number of total transcripts and percentage of expressed mitochondrial RNA in sample cells from the study. (**C**) Expression of the candidate cell-type-specific markers of oocytes (Oo), cumulus cells (CC) and mural granulosa cells (MGC) on violin plots.

Figure S2

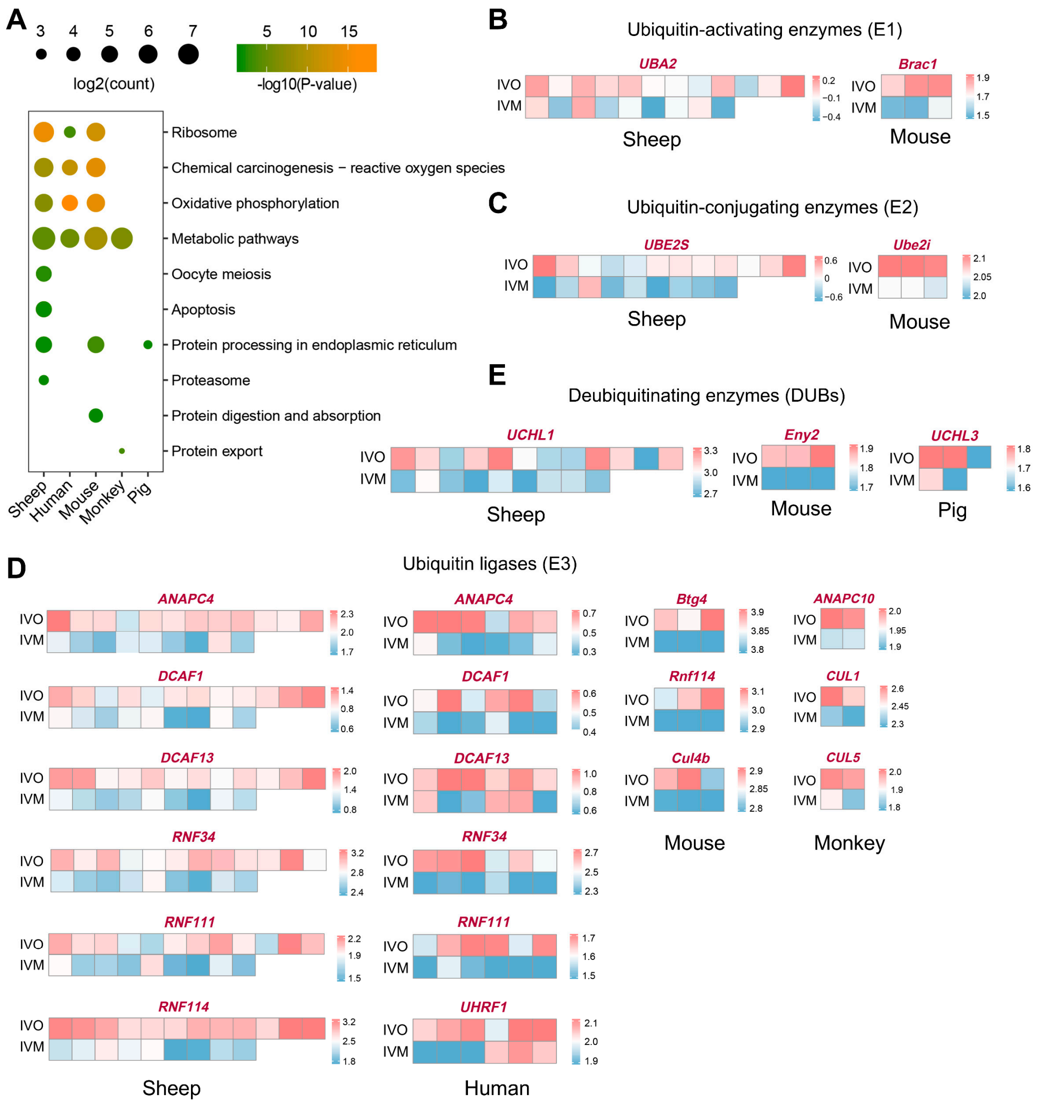

Supplementary Material 3: **Figure S2.** Conserved features of oocyte maturation: comparing *in vivo* and *in vitro* environments across species.

(A) KEGG enrichment of differential genes in oocytes from *in vivo* and *in vitro* matured across different species. (**B-E)** Heatmaps showing scaled expression levels of ubiquitin-proteasome pathway components (E1, E2, E3, and DUBs) across multiple specie.

Figure S3

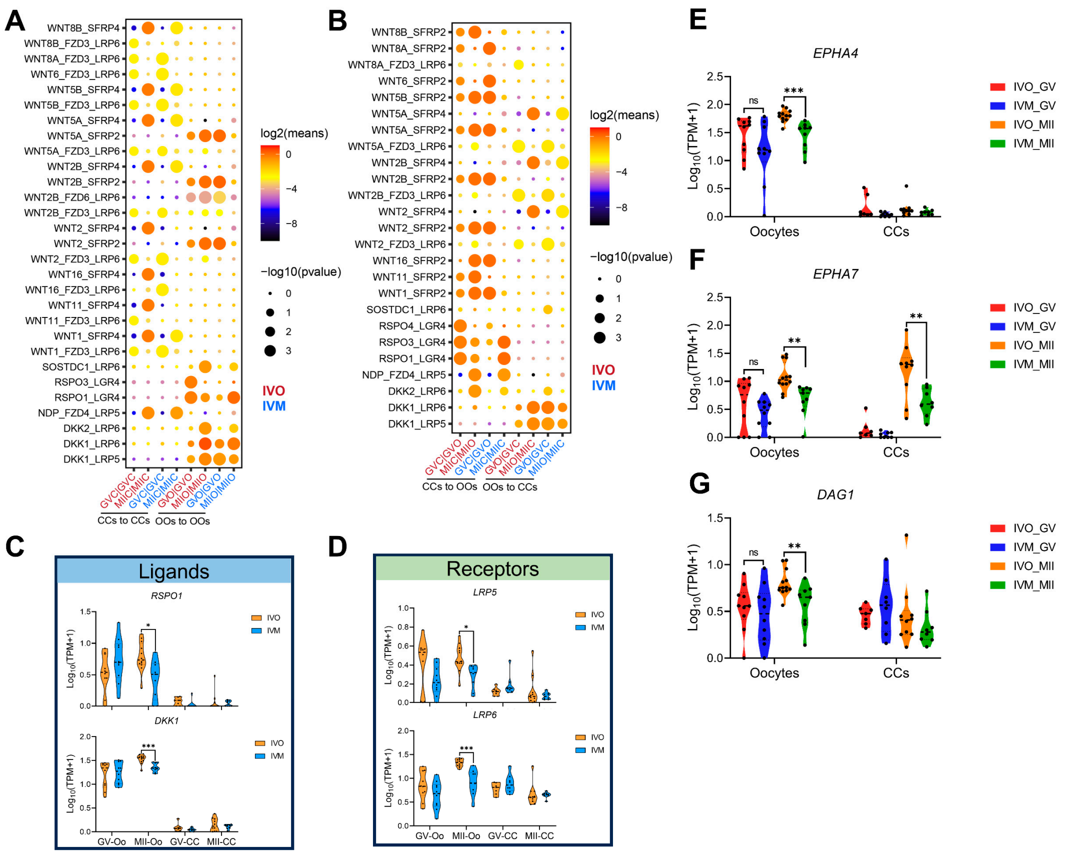

Supplementary Material 4: **Figure S3.** WNT signaling interactions between oocytes and cumulus cells in different maturation environments.

(A-B) CellPhoneDB analysis of WNT signaling interactions between oocytes and cumulus cells (autocrine and paracrine) in different maturation environments. **(C-D)** Violin plots of selected WNT signaling ligand and receptor genes in oocytes and cumulus cells. **(E-G)** EFNA1 receptors (*EPHA4, EPHA7*) and NRXN1 receptors (*DAG1*) expression was shown in violin plots; Statistical significance: * $p < 0.05$, ** $p < 0.01$, *** $p < 0.001$.

Figure S4

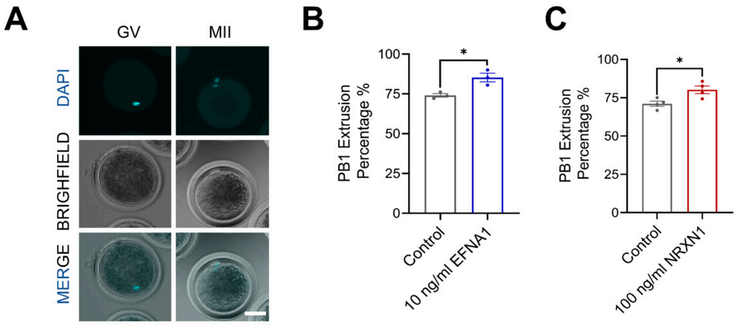

Supplementary Material 5: **Figure S4.** Effects of EFNA1 and NRXN1 on oocyte nuclear maturation. (**A)** The representative images of germinal vesicle (GV) oocyte, metaphase II (MII) oocyte (Scale bar = 50 μm). (**B**-**C)** Individual effects of EFNA1 and NRXN1 on first polar body (PB1) extrusion of oocytes. Statistical significance: * $p < 0.05$.
